# Reducing uncertainty in genetic testing with Saturation Genome Editing

**DOI:** 10.1515/medgen-2022-2159

**Published:** 2022-11-29

**Authors:** Phoebe Dace, Gregory M. Findlay

**Affiliations:** The Genome Function Laboratory, The Francis Crick Institute, 1 Midland Rd, London, United Kingdom

**Keywords:** variants of uncertain significance, functional assays, genotype–phenotype, CRISPR, Saturation Genome Editing

## Abstract

Accurate interpretation of human genetic data is critical for optimizing outcomes in the era of genomic medicine. Powerful methods for testing genetic variants for functional effects are allowing researchers to characterize thousands of variants across disease genes. Here, we review experimental tools enabling highly scalable assays of variants, focusing specifically on Saturation Genome Editing (SGE). We discuss examples of how this technique is being implemented for variant testing at scale and describe how SGE data for *BRCA1* have been clinically validated and used to aid variant interpretation. The initial success at predicting variant pathogenicity with SGE has spurred efforts to expand this and related techniques to many more genes.

## Introduction

Technologies to cost-effectively ascertain human genetic variation have greatly advanced our understanding of genotype–phenotype associations and the molecular basis of human disease. However, for each gene in which variants have been associated with disease it often remains unknown which of *all possible* variants in and near the gene will, indeed, cause disease.

**Figure 1:**
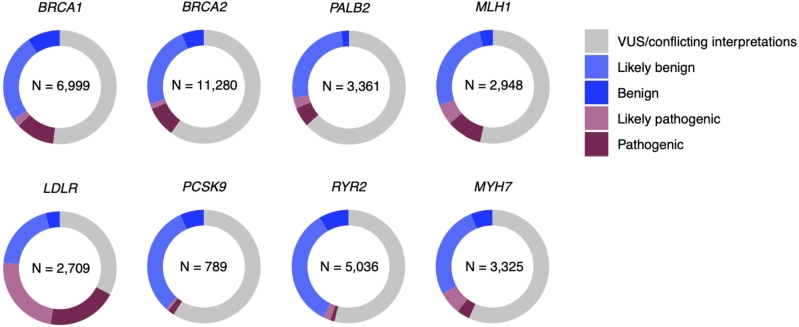
ClinVar variants in commonly sequenced genes. The number of variants reported as of September 2022 is shown for each of eight genes for which secondary findings are returned [37]. Variants of uncertain significance (VUS) or with conflicting interpretations are shown in gray. For most genes, VUS outnumber variants deemed pathogenic or benign.

This is evidenced clinically by large numbers of “variants of uncertain significance” (VUS) observed in commonly sequenced genes (Fig. 1) [1]. Considering nearly all single nucleotide variants (SNVs) compatible with life are predicted to exist in at least one living individual [2], the true number of variants of unknown disease association is far greater than the total number of VUS observed. Furthermore, VUS rates are particularly high in non-European populations, who are poorly represented in many of the largest genetic databases [3].

For many diseases, specific management strategies may improve outcomes once a definitive genetic diagnosis is reached. In the realm of cancer predisposition syndromes, for instance, increased screening and prophylactic procedures can substantially increase overall survival for carriers of pathogenic variants [4]. Additionally, new classes of targeted therapeutics have proven effective for treating cancers of specific genetic etiologies [5], [6], [7]. Therefore, to maximize the value of genetic testing, it is imperative to be able to accurately predict the molecular and phenotypic consequences of any given variant.

This is, however, a monumental challenge. With over 6 million variants observed per whole genome sequenced [8], accurately identifying which variants contribute to each of thousands of monogenic disease phenotypes is hard enough for single patients, let alone whole populations. The magnitude of the challenge posed by rare variant interpretation necessitates leveraging orthogonal strategies. Sampling DNA from more individuals will continue to gradually increase the statistical power of genetic associations. However, for the foreseeable future classical genetics approaches will continue to prove inadequate for enumerating *all* variants that cause disease phenotypes. After all, most variants with phenotypic effects driving monogenic disease are exceedingly rare [9].

When rare variants are encountered in disease genes, computational models to predict their effects are routinely used to aid interpretation (e. g., CADD [10], REVEL [11]). These models, increasingly derived using machine learning [12], [13], [14], typically leverage combinations of sequence conservation and biochemical data to predict deleteriousness. This makes them unmatched in regards to scalability, as virtually every variant in the genome can be assigned a pre-computed score. However, their predictions are typically not accurate enough to be used without additional evidence [15].

## MAVEs test thousands of genetic variants per experiment

To better understand sequence–function relationships, many research teams have contributed to a suite of experimental methods now collectively known as multiplex assays of variant effect (MAVEs) [16]. MAVEs allow thousands of different variants to be assessed in a pooled format to reveal each variant’s functional consequence. The highly scalable nature of MAVEs relieves the bottleneck of testing variants individually. Yet, developing MAVEs that faithfully reflect variant effects on disease processes is challenging, as is translating experimental results into actionable clinical evidence.

Next-generation sequencing (NGS) platforms have enabled exponential growth in human genetic data by drastically reducing the cost of sequencing DNA. Sequencing technology is now used routinely to quantify the relative abundances of different DNA sequences present in complex samples. Powerful methods based on quantification via NGS include single-cell RNA sequencing to measure gene expression in individual cells and CRISPR knockout screens to ask which genes drive cellular phenotypes. A common feature of MAVEs is that they similarly leverage the scalability afforded by NGS for counting DNA molecules, as precise quantification of variants present in experimentally manipulated samples can enable accurate inference of functional effects.

In a typical MAVE experiment, hundreds to hundreds of thousands of variants are generated in a pooled fashion using a strategy such as custom oligonucleotide synthesis or error-prone PCR [17], [18]. This “variant library” is cloned into an expression vector designed such that each variant may influence a specific molecular or cellular phenotype. NGS is used to “read out” the experiment, i. e., to quantify variants’ effects on phenotype by measuring differences in read counts between variants tested. Deep mutational scanning (DMS) is one type of MAVE used to ask how missense variants affect, for instance, protein stability or enzymatic activity [19]. Likewise, massively parallel reporter assays (MPRAs) reveal how variants in regulatory sequences such as promoters or enhancers alter transcript levels [20], [21]. The key to a successful MAVE is being able to quantify each specific variant’s effect with NGS, such that all variants can be tested in a pooled format, thereby drastically reducing cost and processing time.

For a MAVE to aid clinical variant interpretation, the assay must faithfully distinguish variants associated with disease. This can be challenging because pathogenic variants within a single gene often exert effects through diverse molecular mechanisms. Nonetheless, many recent MAVEs have predicted variant pathogenicity with high accuracy [22], [23], [24], [25], suggesting MAVE data will be valuable for classifying new variants.

**Figure 2:**
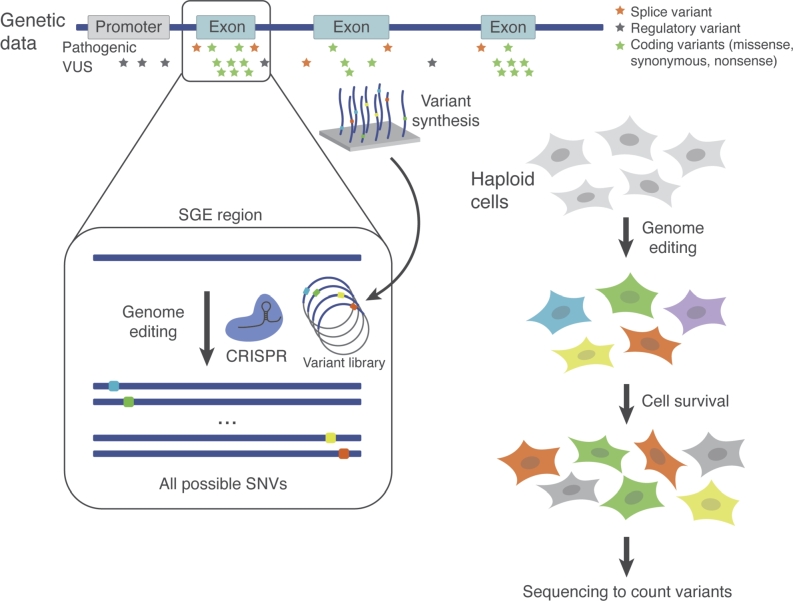
The Saturation Genome Editing method for testing human variants. Variant libraries are designed to include all single nucleotide variants in a genomic region of interest, such as an exon of a gene harboring many VUS. Variants are synthesized in an oligo pool, cloned into a library, and integrated to the genome of human cells with CRISPR technology. In the HAP1 essentiality assay (right), variants are created in a gene required for cell growth. Loss-of-function variants lead to growth defects and are depleted from the population over time. Next-generation sequencing is used to quantify variant abundances at multiple timepoints, such that variant effects can be deduced from sequencing data.

## Developing Saturation Genome Editing to test variants at the endogenous locus

One key limitation of many functional assays is that variants are tested outside their endogenous genomic context. For instance, missense variants are typically expressed in cDNA vectors lacking introns and endogenous regulatory elements. This compaction of sequence space can increase assay throughput but may confound interpretation because pathogenic variants can affect any number of molecular processes involved in gene function (e. g., transcription, RNA splicing, protein folding, protein function). Different variants affecting the same gene may require different assays depending on their mechanism of action, for instance, one assay for splicing and another for enzymatic activity.

To overcome these limitations, we developed Saturation Genome Editing (SGE) as a way to systematically test variants in the context of the human genome [26]. In a typical SGE experiment, all possible SNVs in a short region (up to 150 bp) are tested in a single pool, along with any other variants of interest, such as in-frame insertions and deletions (Fig. 2). Variants are cost-effectively synthesized in oligonucleotide pools, meaning any short variant (<50 bp) can be engineered. The variant library is amplified, cloned into “donor” plasmids to facilitate homology-directed DNA repair (HDR), and introduced to cells using CRISPR gene editing [27]. Each successfully edited cell receives a single variant from the library. By genetically modifying millions of cells at a time, each variant in the library is created many times independently, minimizing potential effects of off-target CRISPR editing.

Once variants are introduced to cells, a functional selection is applied to discriminate variant effects. We initially leveraged the fact that across human cell lines a subset of expressed genes are required for proliferation [28]. We showed that loss-of-function variants in these “essential” genes can be distinguished from functionally neutral variants by performing SGE of the intron lariat debranchase *DBR1* in a haploid human line, HAP1 [29]. Allowing edited cells to proliferate in culture and measuring variant depletion in the cell population over time revealed nonsense variants and missense variants at the enzyme’s active site to be highly deleterious [26]. We also demonstrated that the same experimental workflow could be used to ask specific molecular questions, such as how different variants impact splicing.

## Analyzing thousands of *BRCA1* variants with SGE

We next applied SGE to study variants in *BRCA1*, a tumor suppressor gene emblematic of the challenge posed by clinical variant interpretation. BRCA1 is involved in maintaining genomic stability and DNA repair by homologous recombination and germline mutations leading to loss-of-function cause hereditary breast and ovarian cancer. Like many frequently tested genes, *BRCA1* harbors thousands of VUS, typically rare missense and exon-proximal intronic variants with unknown effects. Accurately identifying pathogenic *BRCA1* variants is crucial for ensuring patients and their families are presented with options to reduce their cancer risk [4].

**Figure 3:**
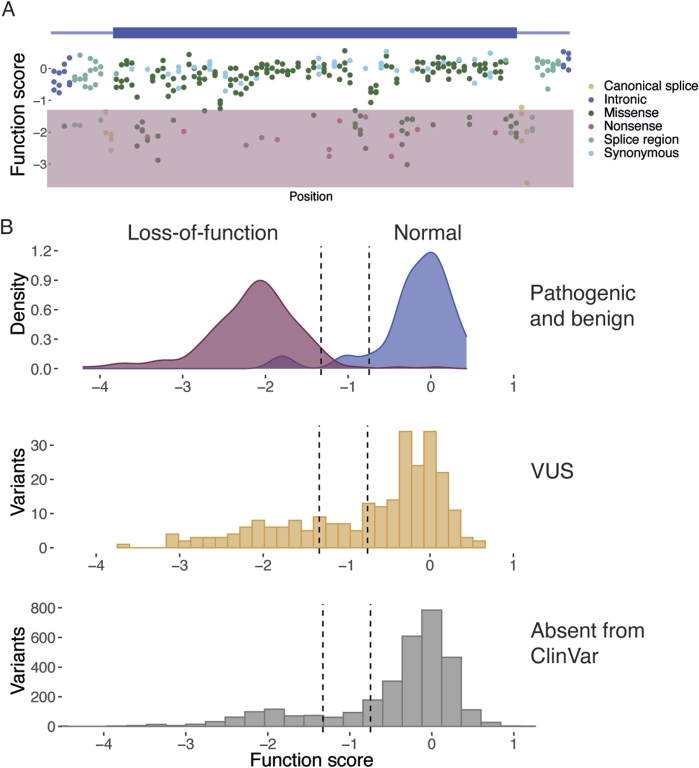
SGE of *BRCA1* improves clinical variant interpretation. SGE was used to produce a variant effect map of BRCA1 RING and BRCT domains, comprising function scores for each of 3,893 SNVs across 13 exons. (A) Function scores for SNVs in a single exonic region (exon 4) are plotted by position. Variants scoring similarly to nonsense variants are deemed loss-of-function variants (shaded). (B) Function scores discriminate known pathogenic variants with >95 % accuracy (top), and can be used as strong evidence for classifying VUS (middle) and variants yet to be reported in ClinVar (bottom). Data from Findlay et al. 2018.

We prioritized assaying all possible SNVs in BRCA1’s RING and BRCT domains, which harbor most of the gene’s established pathogenic missense variants [22]. To do this, we applied an optimized SGE protocol to characterize a total of 3,893 SNVs across 13 exonic regions, culminating in a variant effect map of the two domains (Fig. 3A). Variants such as nonsense variants expected to cause loss-of-function consistently scored lowly, whereas only ∼1 % of synonymous variants proved deleterious to cells. Missense variants tended to either score lowly or have no effect on function, but a small fraction scored intermediately.

## The clinical impact of SGE data

It was initially unclear how useful the *BRCA1* SGE data would be for variant interpretation, chiefly because HAP1 cells had not been used previously as a model for *BRCA1* function and lack clear relevance to breast and ovarian cancer. Therefore, analyzing how accurately the SGE assay adjudicated well-established pathogenic and benign variants was critical for determining utility. Comparing the SGE data for *BRCA1* variants to established interpretations in ClinVar [1] revealed pathogenic variants were identified with >95 % specificity and sensitivity [22] (Fig. 3B). Crucially, this analysis revealed the assay to be highly accurate independent of variant type (e. g., nonsense, missense, synonymous, intronic), owing to the fact variants were edited into the human genome with CRISPR.

Several recent studies have independently validated the utility of SGE data for adjudicating clinically observed variants in conjunction with other lines of evidence. A comprehensive comparison of different BRCA1 assays revealed SGE to be among the most accurate despite having much greater scalability [30]. An analysis of variants observed in a breast cancer cohort revealed women with a VUS defined as loss-of-function by SGE had indistinguishable clinical characteristics to women harboring known pathogenic variants [31]. Another group asked whether SGE data predicted *BRCA1*-related cancer risk in an unbiased cohort of over 92,000 individuals. Indeed, women with variants measured to be loss-of-function had much higher risk of breast and ovarian cancer [32]. Lastly, Fayer et al. found that approximately half of the VUS in *BRCA1* tested via SGE could be clinically reclassified as “likely pathogenic” or “likely benign” [33], illustrating the potential to substantially improve the value of genetic testing for many patients.

## Recent implementations of SGE and future advances

The clinical utility of *BRCA1* SGE data has spurred efforts to apply the method to other disease genes, including *CARD11* and *DDX3X* [34], [35]. In both studies, SGE again proved accurate at predicting disease risk conferred by variants, suggesting simple cellular models may be sufficient for determining variant pathogenicity across a range of genetic disorders. Like *BRCA1*, many other tumor suppressors are essential in the HAP1 line [36], indicating the same assay used for *BRCA1* may prove valuable for resolving variants in many more genes (e. g., *BRCA2*, *PALB2*, *RAD51D*, *RAD51C*) [37]. Indeed, efforts to perform SGE on many more cancer predisposition genes are ongoing [38]. It will remain critical that each new data set for a gene be carefully calibrated against clinical evidence. Towards this end, continued open sharing of genetic data and assay data will be essential for ensuring maximal impact. Initiatives to facilitate sharing include MaveDB [39], CanVIG [40], and the BRCA Exchange [41], and functional data can be both deposited on their own or cited as evidence in ClinVar [1].

While the HAP1 line has proven useful for assessing variant effects in numerous genes, in order to perform different functional assays that require other cell types to reflect biologically meaningful effects, it will be necessary to develop models of physiologically diverse diseases that are amenable to SGE. Haploidization of specific gene loci has been demonstrated as a strategy to allow editing of a single allele, permitting variant interpretation in diploid cell lines [25]. In order to understand the effects of variants on more complex disease processes, for instance developmental disorders, improved tools for generating and tracking variant libraries in organoid systems and model organisms will be required. Applying SGE and similar approaches to study variants with more subtle functional effects, such as those identified by genome-wide association studies, will require further technology development. Assays yielding highly quantitative data on gene expression in cell models which faithfully recapitulate effects of disease-associated noncoding variants will be particularly valuable for this task.

As CRISPR technology continues to improve, the ease of performing SGE in increasingly complex models of disease will inevitably increase. Indeed, newer editing reagents are already allowing more variants to be tested. CRISPR “base editor” screens [42], [43], in which tens of thousands of variants across multiple genes are engineered and assayed simultaneously, have emerged as a means of creating more variants per experiment, albeit with notable trade-offs. Another recent CRISPR-based technology called “prime editing” [44] has proven well suited for achieving saturation mutagenesis of endogenous regions of *NPC1* and *BRCA2* [25]. It is inevitable that larger variant effect maps will be generated in the near future using genome editing methods.

The forthcoming variant effect maps for many disease genes will bring challenges regarding how to best integrate and interpret orthologous data. For instance, different MAVEs covering variants in the same gene may produce contradictory results. In the rare instances where assay data contradict established interpretations from human genetic data, investigating the molecular basis of the discrepancies may prove necessary, and doing so will likely further our understanding of disease mechanisms. For variants that score intermediately in a functional assay, there may not be enough clinical data to calibrate disease risk. Yet over the next decade, having functional data for far more variants than ever before will surely improve efforts to establish more quantitative frameworks for genetic risk that go beyond simple “pathogenic” and “benign” distinctions. Ultimately, large amounts of unbiased functional data will also likely improve computational models of variant effect [45].

## Conclusions

Experimental methods such as SGE that enable high-throughput characterization of human variants may soon bring about an era in which clinical geneticists have prospective functional data on any possible mutation likely to be seen in a disease-associated gene. While the technologies ushering in this new era are still improving, the high clinical utility of recent data sets suggest these methods will prove highly valuable for eliminating the considerable amount of uncertainty that exists in genetic testing today.
